# Tobacco industry pricing strategies in response to excise tax policies: a systematic review

**DOI:** 10.1136/tobaccocontrol-2021-056630

**Published:** 2021-08-09

**Authors:** Zaineb Danish Sheikh, J Robert Branston, Anna B Gilmore

**Affiliations:** 1 Tobacco Control Research Group (TCRG), Department for Health, University of Bath, Bath, UK; 2 School of Management, University of Bath, Bath, UK

**Keywords:** taxation, price, tobacco industry

## Abstract

**Objective:**

To explore what is known about the tobacco industry’s (TI) price-based responses to tobacco excise tax policies and whether these vary by country income group using a systematic review.

**Data sources:**

Studies assessing TI pricing tactics were identified via searches of five online databases using a combination of search keywords.

**Study selection:**

Inclusion criteria were applied by two reviewers independently who screened all search results (titles and abstracts) for possible inclusion. They identified 37 publications that reported TI pricing tactics.

**Data extraction:**

Study details were tabulated, and information was extracted on the country income group, population characteristics, excise tax structure, and pricing strategies.

**Data synthesis:**

Of the 37 publications identified, 22 were conducted in high-income countries, while 15 covered low-income and middle-income countries (LMICs). Major pricing strategies employed were: differentially shifting taxes between products (35 studies); launching new brands/products as pathways for downtrading (six studies), product promotions and different prices for the same products for different customers (six studies); price smoothing (two studies); and changing product attributes such as length/size of cigarettes or production processes (three studies).

**Conclusions:**

While there is limited evidence to fully ascertain industry responses to tax increases, this review suggests that the TI widely uses a multitude of sophisticated pricing strategies across different settings around the world with the intention of undermining tax policies, thereby increasing tobacco consumption and maximising their profits. There is a need for further research in this area especially in LMICs so that effective policy responses can be developed.

## Introduction

Excise taxes on tobacco products are the most powerful and cost-effective tobacco control measure, since the resulting higher prices both reduce consumption and expand government revenues.^1–3^ Higher prices discourage smoking among current smokers and prevent initiation, especially by the price sensitive young.^4^ The WHO’s Framework Convention on Tobacco Control (FCTC) recognises the importance of this policy in Article 6 and calls on governments to implement tax/price measures to reduce tobacco use.^5^ However, the success of taxes is largely dependent on the extent to which the industry passes on tax increases and uses strategies to mitigate their impact.^6 7^

An examination of internal tobacco industry (TI) documents revealed their knowledge of the influence of price on cigarette demand and consumer behaviour.^8–12^ Furthermore, the oligopolistic nature of tobacco markets with limited competition gives them significant pricing power, especially since demand for their products is relatively inelastic, resulting in inordinate profitability.^8 13 14^ This profit and pricing power therefore gives the TI options when it comes to responding to tax increases.

However, studies exploring industry price-based responses to tax increases are a relatively recent area of academic enquiry and have tended to focus on particular countries, notably high-income countries (HICs). As such, little is known about overall behaviours or trends especially across country income groups. Since pricing tactics impact the effectiveness of tobacco taxes, they are important for all countries to understand, particularly for low-income and middle-income countries (LMICs). Global tobacco consumption has become increasing skewed towards these countries where, with a few exceptions, cigarette affordability has generally increased,^15–19^ giving the TI even greater potential to use price to undermine tax increases.

Given the emerging nature of research in this area, it is not yet clear if the current evidence on industry pricing strategies has identified tactics that are consistently used across differing countries, country income groups, or other contexts. For example, industry pricing will likely vary as it shifts from demand maximisation to profit maximisation.^13 20^ Furthermore, evidence suggests that cigarette price differentials (difference between the cheapest and most expensive brands) are generally larger in LMICs than in many HICs, which allows the industry greater pricing freedom.^21 22^ There are therefore significant uncertainties in regards to the industry’s response to tax and associated pricing policies.

Consequently, this paper aims to undertake a systematic review of the literature on the TI’s responses to tax increases in order to explore the range of pricing strategies they adopt to undermine their impact and specifically whether these vary by country/income-group/overtime/across price segments/products. Understanding these strategies is essential for developing appropriate countermeasures and hence the most effective tobacco taxation policies. The paper does not intend to examine wider industry pricing strategies, such as their arguments against higher taxation, including the threat of illicit trade, as these have been examined in detail elsewhere including via systematic review.^23–26^

## Methods

A systematic review methodology was adopted, following the Preferred Reporting Items for Systematic Reviews and Meta-Analyses (PRISMA) reporting guidelines.^27^ A full protocol for this systematic review was developed and is registered with the PROSPERO international prospective register of systematic reviews (ID: CRD42021234336) at http://www.crd.york.ac.uk/PROSPERO.

### Search strategy

We aimed to identify all independent empirical studies published in academic journals on industry pricing strategies in response to excise taxes. We therefore developed a comprehensive search strategy with the assistance of a reference librarian. Systematic searches were undertaken in five electronic databases (MEDLINE, Web of science, Embase, Scopus, and ScienceDirect), which were then verified by the main findings of the same searches in PubMed Central and Google Scholar. The following search string was applied with minor adaptations for the requirements of each database:

(“Tobacco” OR “cigar*” OR “smok*“) AND (“Tobacco company” OR ‘“transnational tobacco company” OR “transnational*” OR “TTC” OR “TTCs” OR “industr*” OR “manufacturer*“) AND (“taxation” OR “tax” OR “excise” OR “price” OR “pricing”) AND (“strateg*” OR “tactic*” OR “polic*” OR “intervention” OR “influence”)

Titles and abstracts of potentially relevant studies were independently screened for relevance against the eligibility criteria by one of two reviewers who also both screened a 10% sample of the other’s exclusion decisions to ensure consistency. Following preliminary review, full texts of all studies that were found to be possibly relevant were then obtained and assessed independently by two reviewers according to the eligibility criteria. Any disagreements during the entire selection process were resolved through examination of the full manuscript, discussion and, where necessary, consultation with the third author.

### Eligibility criteria

All searches were conducted between April and September 2020, and the search was not restricted by study design or year of publication. Similarly, no language or geographic restrictions were used. We included original studies that are primarily quantitative and/or qualitative peer-reviewed research articles featuring industry pricing tactics in response to tax policies. We excluded duplicate reports of the same study, brief reports that were only abstracts and conference abstracts. Unless price was mentioned, articles that focused on other TI strategies (such as lobbying/marketing/advertising, etc) were also excluded.

### Data extraction

Data from each study were extracted by one reviewer and verified by at least one other member of the author team. Studies were tabulated by country and country income groups: high, upper middle, lower middle, and low income using the World Bank’s (WB) classifications with the year of the study also recorded.^28^ We identified not just the year of publication but the year(s) the data covered. We sought additional data from the time of the different studies to contextualise our findings: information on tobacco taxes, including tax structure, excise tax as a proportion of price, and reliance on specific tax were taken from the WHO reports on Global Tobacco Epidemic, and gross domestic product (GDP) per capita at purchasing power parity (PPP) was taken from The WB’s statistics.^29^

All studies were reviewed in depth by all the authors who met regularly to review and discuss results and categorisation of industry pricing strategies until final classification and evidence tabulation had been agreed. Strategies not explicitly examined were reported as ‘not examined (N/E)’ but where examined and found not to occur, were recorded as ‘absent’.

### Data synthesis

We did not formally assess the methodological quality of reviewed articles because the goal of this review was not to assess the robustness of the evidence but rather to examine the breadth of strategies seemingly employed by the TI in different countries. However, we remained mindful of study quality when reading each paper in case any featured an obvious weakness or error that we wanted to highlight (none did). A narrative review is conducted and reported as wide heterogeneity in study design/settings/populations/indicators/outcome measures assessed among the included studies precluded a meta-analysis.^30 31^ However, where possible, quantitative results are compared between studies.

## Results

### Study selection

**Figure 1:**
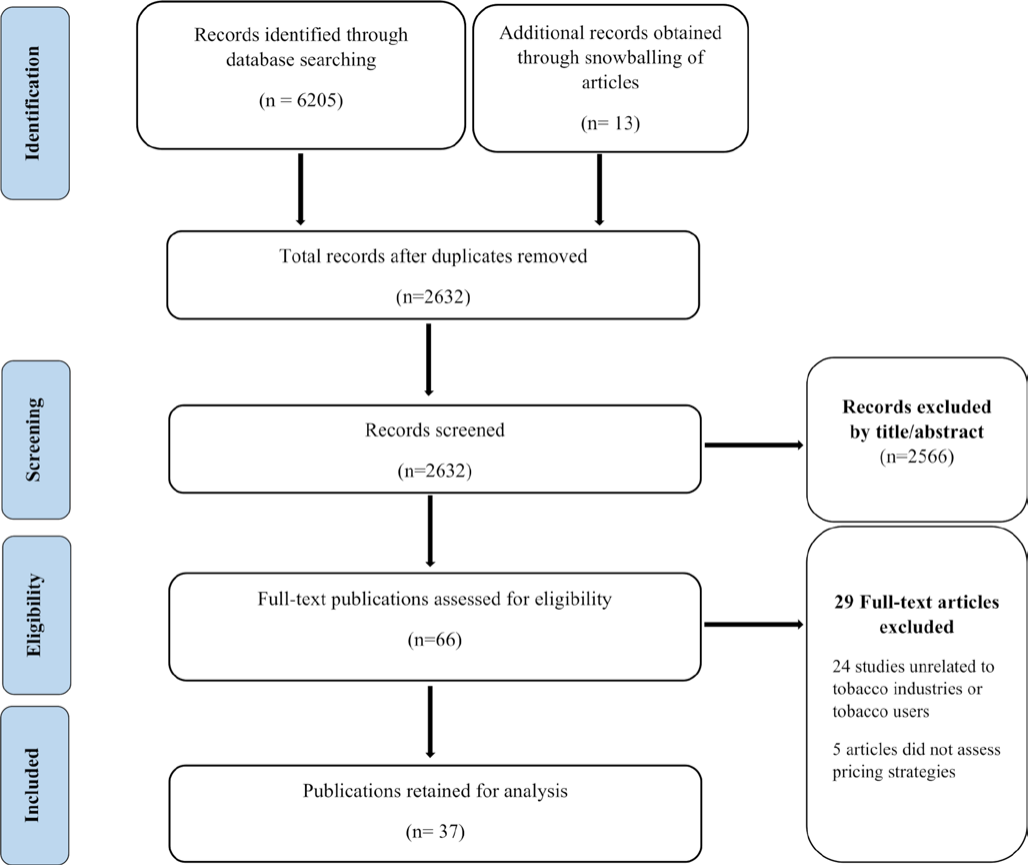
PRISMA study selection flow chart here. PRISMA, Preferred Reporting Items for Systematic Reviews and Meta-Analyse.

An initial search of the five databases located 6205 potential publications (which was then verified with the searches in Google Scholar and PubMed). A further 13 abstracts that were not in the search results, but which potentially met the inclusion criteria, were identified through forward and backward snowballing. The records were stored in a reference management system (Endnote), which facilitated ease of screening for duplicates and organisation of the final set of abstracts leaving 2632 documents. Of these, 2566 were excluded based on title and abstract screening. The inclusion and exclusion criteria were then applied to the remaining 66 publications, bringing the number of papers to 37 (figure 1 shows the PRISMA study selection flow chart).

### Characteristics of the included studies

The sample of 37 articles were all published between 1996 and September 2020. Twenty-two were conducted in HICs, 15 in LMICs, and none covered a low-income country (table 1). All 37 focused on pricing strategies for factory-made (FM) cigarettes, while five also briefly discussed roll-your-own tobacco (RYO). The majority of studies used commercial pricing data obtained either from governmental sources or independent market research databases (eg, Euromonitor/Nielsen), while a few used data from surveys of retailers or consumers (eg, ITC survey). We identified six broad pricing strategies that the TI employs: differentially shifting taxes; price smoothing; shrinkflation; changing product attributes or production processes; price discrimination and price related promotions; and introducing new brands/segments/products as pathways for downtrading (see table 2 for definitions). While these tactics seem to vary in relation to the income level of countries, we did not find any correlation with the type of excise taxes or taxation structures.

**Table 1:** Summary of studies concerning tobacco industry pricing strategies

Countries	Type of excise tax at the time of study	Excise tax proportion of price (%) at the time of study	Taxation system at the time of study	Greater reliance on specific tax (in mixed excise regime)	Studies	Year of publication	GDP per capita, PPP at the time of study (current international $)	TI pricing strategies							
								Differentially shifting taxes			Introducing new brands/segments/products as downtrade pathways	Changing product attributes or production processes	Price discrimination and price related promotions	Price smoothing	Shrinkflation
								Overshifting	Undershifting	Overall pattern					
**High-income countries**															
**UK**	Mixed excise	61.8	Uniform	Yes	Understanding TI pricing strategy and whether it undermines tobacco tax policy: the example of the UK cigarette market.^41^	2013	39 970.9	Taxes are overshifted in all brand segments although at a lower rate for the ultra-low price segment	In March/April each year, the industry undershifts tax increase on ultra-low price brands.	Overshifting	N/E	N/E	N/E	N/E	N/E
	Mixed excise	62.72	Uniform	Yes	TI strategies undermine government tax policy: evidence from commercial data.^42^	2018	46 867.9	Overshifting in higher segments	Undershifting in lower segments	Overshifting	Creation of new FM and RYO segments and launch of cheap products	Brands were repriced from high to low segment	TI introduced new ultra-low price category and kept its price constant via price discrimination	TI uses price smoothing	Amount sold per pack was reduced
	Mixed excise	62.72	Uniform	Yes	UK tobacco price increases: driven by industry or public health?^43^	2019	48 709.7	Overshifting taxes for both FM and RYO cigarettes	Undershifting on cheaper product	Overshifting	N/E	N/E	N/E	N/E	N/E
	Mixed excise	62.72*	Uniform	Yes	Quantile regression of tobacco tax pass-through in the UK 2013–2019. How have manufacturers passed through tax changes for different tobacco products?^44^	2020	48 709.7†	Overshifting across the price distribution for both FM and RYO	–	Overshifting	N/E	N/E	N/E	N/E	N/E
	Mixed excise	62.72*	Uniform	Yes	Impact of tobacco tax increases and industry pricing on smoking behaviours and inequalities: a mixed-methods study.^45^	2020	48 709.7†	Real prices of premium FM and RYO packs increased	Undershifting on subvalue packs and RYO tobacco packs	Overshifting	Creation of new FM and RYO segments and launch of cheap products including cigarillo	Brands were repriced from high to low segment	TI introduced new ultra-low price category and kept its price constant via price discrimination	TI uses price smoothing	Smaller packs (17–19 sticks) were introduced
**Ireland**	Mixed excise	61	Uniform	Yes	The Irish TI position on price increases on tobacco products.^46^	2011	44 855.3	TI persistently raised tobacco prices by overshifting tax increase	Absent	Overshifting	N/E	N/E	N/E	N/E	N/E
**USA**	Specific excise†	N/A	Uniform	–	Do cigarette producers price-discriminate by state? An empirical analysis of local cigarette pricing and taxation.^32^	1996	29 967.7	State taxes are overshifted	Absent	Overshifting	N/E	N/E	Cigarette producers do price-discriminate by state	N/E	N/E
	Specific excise†	31.55‡	Uniform	–	The incidence of tobacco taxation: evidence from geographic micro-level data.^33^	2008	48 382.5	Tobacco tax was overshifted to consumers	Absent	Overshifting	N/E	N/E	N/E	N/E	N/E
	Specific excise†	37.73‡	Uniform	–	The heterogeneous geographic and socioeconomic incidence of cigarette taxes: evidence from Nielsen Home scan Data.^52^	2012	51 610.6	Absent	Taxes are less than fully passed through to consumer prices	Undershifting	N/E	N/E	N/E	N/E	N/E
	Specific excise†	37.73‡	Uniform	–	The effect of cigarette taxation on prices: an empirical analysis using local-level data.^34^	2012	51 610.6	Cigarette excise taxes are overshifted at both the state and local levels of government	N/E	Overshifting	N/E	N/E	N/E	N/E	N/E
	Specific excise†	37.73‡	Uniform	–	Who pays cigarette taxes? The impact of consumer price search.^35^	2013	53 117.6	Taxes are overshifted to smokers of light cigarettes	Taxes undershifted to heavy smokers, those who buy cartons and consumers who live near the border of a lower tax state	Overshifting	N/E	N/E	Discounts on buying cartons	N/E	N/E
	Specific excise†	37.38‡	Uniform	–	Does every US smoker bear the same cigarette tax?^36^	2014	55 047.7	Overshifting for smokers of premium brands who purchased by pack outside Indian reservations	Excise tax was undershifted to some smoker’s price-minimising strategies	Overshifting	N/E	N/E	Price reductions on purchase of cartons, buying on Indian reservations or buying generic brands	N/E	N/E
	Specific excise†	37.38‡	Uniform	–	Continued implications of taxing RYO tobacco as pipe tobacco in the USA.^50^	2014	55 047.7	N/E	N/E	N/E	N/E	Tobacco manufacturers repackaged roll-your-own tobacco as pipe tobacco	N/E	N/E	N/E
	Specific excise†	37.81‡	Uniform	–	Tobacco product prices before and after a state-wide tobacco tax increase.^89^	2016	57 927.5	Overshifting of the tobacco tax to consumers	Absent	Overshifting	N/E	N/E	N/E	N/E	N/E
	Specific excise†	37.81‡	Uniform	–	Exploring impacts of taxes and hospitality bans on cigarette prices and smoking prevalence using a large dataset of cigarette prices at stores 2001–2011, USA.^37^	2017	59 957.7	N/E	Cigarette excise taxes were undershifted and pass-through rates were slightly higher in areas with hospitality bans	Undershifting	N/E	N/E	N/E	N/E	N/E
	Specific excise†	37.76‡	Uniform	–	Mind the gap: changes in cigarette prices after California’s tax increase.^38^	2019	65 280.6	Overshifting of tax across all brands, greater at higher price points (more for ultra-premium than premium and for premium than value brand)	Undershifting was evident in neighbourhoods with more African-American and Hispanic residents	Overshifting	N/E	N/E	After tax increase, prices were significantly discounted, and more stores advertised a discount on cigarettes	N/E	N/E
	Specific excise†	37.76‡	Uniform	–	TI promotions and pricing after tax increases: an analysis of internal industry documents.^39^	2019	65 280.6	Tobacco companies overshifted tax after the US federal tax doubled in 1983	Lowered manufacturer price to undershift smaller tax increases	Both	N/E	N/E	Increased promotions at tax differential stores/border stores after tax increases in neighbouring states	N/E	N/E
**Europe (23 countries**)	Mixed excise	57% excise tax rate on the most popular price category	–	Yes	An analysis of TI pricing strategies in 23 European Union countries using commercial pricing data.^53^	2019	–	N/E	Undershifting in cheap segments of cigarette market. TI maintains or increases price gap.	N/E	N/E	N/E	N/E	N/E	N/E
**Spain**	Mixed excise	64.1	Uniform	No	The Spanish tobacco tax loopholes and their consequences.^51^	2012	31 720.1	N/E	N/E	N/E	TI marketed cheaper alternative tobacco products	N/E	N/E	N/E	N/E
**Czech Republic**	Mixed excise	60.9	Uniform	Yes	TI manipulation of tobacco excise and tobacco advertising policies in the Czech Republic: an analysis of TI documents.^12^	2012	29 047.2	The TI is overshifting tax increases	Absent	Overshifting	N/E	N/E	N/E	N/E	N/E
**New Zealand**	Specific excise	64.16*	Uniform	–	The impact of an increase in excise tax on the retail price of tobacco in New Zealand.^47^	2015	37 475.1	Overshifting tax on premium, mainstream and RYO brands	Undershifting excise tax on the budget brand	Both	N/E	N/E	N/E	N/E	N/E
**Taiwan**	Mixed excise	48	Tiered	No	Exploiting a low tax system: non-tax-induced cigarette price increases in Taiwan 2011–2016.^54^	2019	50 592.5	As a result of low and unchanged tobacco taxes tobacco companies overshifted to increase the real price of cigarettes	Absent	Overshifting	N/E	N/E	N/E	N/E	N/E
**Total HICs (22 studies)**								**17**	**12**		**3**	**2**	**6**	**2**	**2**
**Upper middle income countries**															
**South Africa**	Specific excise	32.44	Uniform	–	Industry responses to the tobacco excise tax increases in South Africa.^66^	2006	10 518.4	Between 1990 and 2005, the industry increased its real retail rate by more than the increase in the real excise tax to increase its revenue	Absent	TI overshifted between 1990 and 2005	N/E	N/E	N/E	N/E	N/E
	Specific excise	40.12	Uniform	–	The effect of excise tax increases on cigarette prices in South Africa.^55^	2017	12 703.4	Between 1994 and 2010, the TI increased retail price of cigarettes twice a year: in March in response to the excise tax increase, and in July/ August largely independent of the price.	Since 2010, the excise tax was substantially undershifted	Undershifting	N/E	N/E	N/E	N/E	N/E
**Mexico**	Mixed excise	52	Uniform	No	Self-reported price of cigarettes, consumption and compensatory behaviours in a cohort of Mexican smokers before and after a cigarette tax increase.^56^	2010	15 260.6	N/E	Tobacco taxes undershifted on cheaper national brands	Undershifting	N/E	N/E	N/E	N/E	N/E
	Mixed excise	52.8	Uniform	No	Tax, price and cigarette brand preferences: a longitudinal study of adult smokers from the ITC Mexico Survey.^57^	2013	17 373.8	N/E	Tobacco taxes undershifted on national/discount brands	Undershifting	N/E	N/E	N/E	N/E	N/E
**China**	Mixed excise	26.2	Tiered	No	Can increases in the cigarette tax rate be linked to cigarette retail prices? Solving mysteries related to the cigarette pricing mechanism in China.^65^)	2012	11 197.5	Absent	Excise tax increase in China does not result in increase in retail price of cigarettes	Undershifting	N/E	N/E	N/E	N/E	N/E
**Thailand**	Mixed excise	N/A	Tiered	Yes	Thailand: winning battles, but the war’s far from over.^61^	2000	7302.8	Absent	TTCs have been absorbing tax increases	Undershifting	Introduced cheaper brands	N/E	N/E	N/E	N/E
**Turkey**	Mixed excise	66.11	Uniform	No	Antitobacco control industry strategies in Turkey.^60^	2018	28 139.1	N/E	In 2011, TTCs undershifted taxes to reduce the amount consumers had to pay	Undershifting	Manufactured slim, super-slim cigarettes and flavoured cigarettes and TI manufactured more varieties of products	N/E	N/E	N/E	N/E
**Mauritius**	Specific excise	70.50	Uniform	–	TI tactics in response to cigarette excise tax increases in Mauritius.^64^	2019	23 942.1	TI overshifted the tax increase on premium and popular brand cigarettes	Substantially undershifted tax increase for economy cigarettes	Both	N/E	N/E	N/E	N/E	N/E
**Lower middle income countries**															
**Ukraine**	Mixed excise	54	Uniform	Yes	Economic and public health impact of 2007–2010 tobacco tax increases in Ukraine.^49^	2011	8218.8	Larger tax increases led to overshifting of taxes	Smaller tax increases were undershifted	Both	N/E	N/E	N/E	N/E	N/E
**Indonesia**	Specific excise	44*	Tiered	–	The tobacco excise system in Indonesia: hindering effective tobacco control for health.^69^	2009	8021.7	Absent	Excise tax is being absorbed by firms rather than being passed on to consumers	Undershifting	N/E	Firms reduced their production levels to fall within lower tax brackets	N/E	N/E	N/E
	Specific excise	49.45	Tiered	–	Firms’ performance under a different cigarette tax system: empirical evidence from Indonesian cigarette manufacturing firms.^58^	2018	11 646.4	N/E	There is presence of tax undershifting	Undershifting	N/E	N/E	N/E	N/E	N/E
	Specific excise	49.45	Tiered	–	Cigarette prices in a complex cigarette tax system: empirical evidence from Indonesia.^59^	2019	12 301.7	Absent	Tax is partially passed on to consumers	Undershifting	N/E	N/E	N/E	N/E	N/E
**Pakistan**	Specific excise	45.79	Tiered	–	Smoke screen: estimating the tax pass-through to cigarette prices in Pakistan.^63^	2016	4410.4	N/E	Taxes are undershifted, more for budget and midrange brands as compared with premium brands	Undershifting	N/E	N/E	N/E	N/E	N/E
**Bangladesh**	Ad valorem excise	56	Tiered	–	A decade of cigarette taxation in Bangladesh: lessons learnt for tobacco control.^62^	2019	4950.7	Increased prices at the high end of the market	Lower rate of excise tax for the low-price tier incentivised the expansion of the market for low-price cigarettes	Undershifting	Downward brand substitution (from high to low price cigarettes). Upward product substitution (from bidis to low-priced cigarettes),	N/E	N/E	N/E	N/E
					TI pricing undermines tobacco tax policy: a tale from Bangladesh.^20^	2020*	4950.7	Overshifting for brands at high end of the tiered system	Undershifting for brands in the low tiers	Undershifting	N/E	N/E	N/E	N/E	N/E
**Total LMICs (15 studies**)								**6**	**14**	-**--**	**3**	**1**	**0**	**0**	**0**
**Total (37 studies**)								**23**	**26**	-**--**	**6**	**3**	**6**	**2**	**2**

*Excise tax proportion of price (%) quoted for the previous year due to data availability.

†Data for GDP per capita, PPP at the time of study (current international $) are presented for 2019 as 2020 data were unavailable.

‡Cigarettes in the USA are taxed at both the federal and state levels, so taxes will vary within country.

FM, factory made; HICs, high-income countries; RYO, roll-your-own; TI, tobacco industry.

**Table 2:** Description of pricing strategies

Pricing strategy	Description
Differential shifting of taxes	Taxes on tobacco products may be shifted to different extents: Overshifting takes place when the TI increases the price of products above that required by the tax increase. The burden of the tax increase (and more) falls entirely on the consumers rather than the producers. Undershifting occurs when the industry absorbs (to some extent) tax increases, thus delaying/preventing the intended tobacco price rises. In this scenario, the cost of the tax increase is at least partially borne by the producers.
Introducing new brands/segments/products	The industry introduces new and cheaper factory made (FM) and roll-your-own products (RYO), including cheaper variants of existing products and potentially new price segments, to increase the opportunities for smokers to down-trade instead of quitting. This strategy helps retain customers who no longer want, or are unable, to pay for higher priced products. Simultaneously, they retain higher priced offerings that allow the industry to profit from those who are willing to pay higher prices for ‘luxury’ brands/products.
Price discrimination and price related promotions	Selling the same product at different prices to different customers, often through targeted price-related promotions, can preserve affordability of products across all income groups following a tax increase. This helps to prevent price-sensitive users from quitting or reducing consumption, ensures potential new customers are not deterred by high prices, but allows the industry to take advantage of those less sensitive to price.
Price smoothing	The industry prevents any sudden jumps/increases in price the consumer would face following tax rises by smoothing that increase throughout the year by employing smaller, more frequent increase in prices. This minimises the public health impact of tax increases by ensuring that smokers never face a sudden, large, potentially quit inducing price increase.
Shrinkflation	Varying the numbers of cigarettes per pack. The industry reduces the number of sticks in a pack from (for example) 20 to 19, 18 or even 17 sticks, or weight of tobacco per pack, to disguise price rises and prevent purchase costs of a packet of tobacco being tipped over certain psychological levels. The higher cost per cigarette does not become immediately obvious to most smokers.
Changing product attributes or production processes	Complex tobacco tax structures that levy different tax rates based on different characteristics (eg, length, weight, price, or product type) mean the tobacco industry can exploit different tax classifications by changing physical product attributes or production methods to achieve classifications with lower tax rates.

TI, tobacco industry.

#### TIs pricing strategies

#### Differential shifting of taxes

The most frequently employed strategy identified through this review was the differential shifting of taxes. The overall pattern found in HICs is overshifting (seen in 15 out of 22 studies) with studies from the USA,^32–40^ UK,^41–45^ Ireland,^46^ Czech Republic,^12^ New Zealand^47 48^ and Ukraine^49^ highlighting this behaviour. The TI in these countries persistently raised tobacco prices by overshifting tax increases on tobacco products at the higher end of the market but undershifted on products in lowest segments to keep their prices cheap. This resulted in an increasing price gap between premium and budget cigarettes as well as the range of prices available within each price segment. A few exceptions to this overall trend were studies where shifting of taxes was: not examined^50 51^; examined but found to be both over and undershifted^39 47^; and undershifted (two from the USA, one from Europe).^37 52 53^ Both these US studies used product level consumer data: namely Nielsen Homescan data for 2006–2007,^52^ and cigarette pricing data from chain supermarkets and drug stores between 2001 and 2011,^37^ and both found that tax increases were only partially passed onto consumers. The study from Europe used Euromonitor passport data covering 23 countries between 2006 and 2017, and found that tax increases were selectively undershifted in the cheap cigarette price segment, whereas fully shifted in the expensive category.^53^

The evidence obtained also suggests that a low and static tax environment enabled overshifting of taxes and allowed the industry to adopt strategic approaches for expanding their markets and generating considerable growth in total revenues. For example, a study from Taiwan shows that between 2011 and 2016 taxes levied on tobacco products remained unchanged, which prompted all the major companies to use aggressive pricing and segmentation strategies in order to maintain customer loyalty while making profits.^54^ However, the industry’s behaviour of overshifting is remarkably similar even in countries where prices of the cheapest segments are relatively high such as the UK and USA.

In contrast to HICs, the predominant pattern in LMICs (11 out of 15 studies) over the years covered (2000–2019) was undershifting. This was observed in 8/10 countries (South Africa,^55^ Mexico,^56 57^ Indonesia,^58 59^ Turkey,^60^ Thailand,^61^ Bangladesh,^62^ Pakistan^63^ and Mauritius^64^). The study authors frequently highlighted that in these countries undershifting does not signify the TI’s lack of scope to overshift but its deliberate choice not to as the primary purpose in these economies seems to have been market expansion as opposed to profit maximisation. Hence, tax increases are mostly absorbed for brands across all price categories.^64^ The pattern varied slightly in China, which is an unusual market given its state-owned tobacco monopoly and its role in setting both prices and taxes. Between 2009 and 2015, the monopoly decided against increasing cigarette prices, which led to reduced profit margins for the industry with no changes in the rates of cigarette consumption in light of constant prices.^65^ Similarly, there is evidence of the pattern changing overtime in South Africa: from overshifting of taxes to undershifting. Between 1990 and 2005, the industry increased its price by more than the increase in taxes in order to increase revenues, which resulted in a positive impact on tobacco control as it reduced cigarette consumption. This prompted the industry to change focus from increasing prices to maintaining market size, resulting in lower rate of decrease in tobacco consumption since then.^66^

Two LMICs (Mauritius^64^ and Ukraine^49^) showed evidence of both overshifting and undershifting of taxes. In Mauritius, excise policies have been in line with the international best practices,^67^ and nominal taxes were increased six times between 2008 and 2018. However, the TI still managed to undermine the policy by adopting a multifaceted pricing strategy of overshifting on premium and popular brands while substantially undershifting on economy cigarettes.^64^ Thus, this selective overshifting and undershifting increased price gaps and product segmentation, a scenario that was also observed in Bangladesh and Mexico.^20 57^ Similarly, the study from Ukraine discovered that smaller tax increases were absorbed in 2007–2008, while larger surges in tax rates forced the tobacco manufacturers to raise their prices in order to maintain profits in the subsequent year.^49^

#### Introducing new brands/segments/products as downtrade pathways

Existing evidence indicates that where multiple products are available in a market, the increase in taxes on one product (usually cigarettes) can encourage smokers to downtrade to cheaper alternatives.^42 51 61 68^ Six identified studies (three each from HICs and LMICs) showed that tobacco manufacturers introduced new brands/segments/products as cheaper substitutes, presumably to make sure cigarette smokers do not quit as taxes cause prices to rise. In the UK,^42 45^ Spain,^51^ and Thailand,^61^ the TI launched new economy products and brands of both FM and RYO. Similarly in other countries, new RYO products and new variants of previous FM brands or new subeconomy brands were launched in the subeconomy sector aimed at women and younger people.^45 61^ Moreover, in the UK both FM and RYO brands were downgraded from a higher to a lower segment category by cutting their price.^45^

A study examined the supply-side factors in Bangladesh’s cigarette market between 2006 and 2017, and noted patterns of change in consumers’ and producers’ behaviour.^62^ The widening price differential among brands led to brand substitution from higher price to low-price cigarettes. Furthermore, income growth and shifting preferences led to product substitution from bidis to FM even though the price of bidis remained unchanged during the study. The TI, therefore, created two different pathways to aid its profitability. By offering low-priced cigarettes, they generated new sales by encouraging a shift from bidis (which are generally independently made and very low priced). Low-priced FM also offered premium FM smokers a cheaper option instead of quitting, thereby helping the industry to maintain sales volumes.

#### Changing product attributes or production processes

In an attempt to exploit the different approaches/levels to taxing different products, the industry may reclassify its products, change their physical attributes or reduce its production levels, so they may fall within lower tax brackets/categories. Three studies (from the UK,^42^ the USA, and Indonesia^69^) indicate that the TI modified its products in order to avoid higher taxes and the resulting impact on sales/profits. However, evidence of this pricing strategy was far less than other tactics, suggesting that either it is not popular or has not been widely explored. Examples include the UK, where cigars were subject to relatively lower axation than cigarettes and exempt from some of the restrictions covering all of the European Union (EU). Consequently, the TI took advantage of these loopholes in legislation by launching economy cigars and low-priced cigarette-like filtered cigarillo products from 2010, including under existing cigarette brand names from 2020.^45 70^ Similarly, in the USA from 2009 to 2013, tax on RYO tobacco was more than that for pipe tobacco so the industry relabelled the former as pipe tobacco reducing its tax liability.^50 71^ As a result, sales of RYO tobacco fell while sales of pipe tobacco increased.

The review illustrates that a complex taxation system such as a tiered system with differential tax rates for each type of product further favours this tactic. For example, the studies covering Indonesia highlighted its elaborate multitiered tax system that varied by type of product (cigarette/kretek), mode of production and manufacturing facility, and favoured smaller scale production facilities. Furthermore, up to a 2009 rule change, tobacco companies had a tax incentive to split production between large numbers of small-scale producers.^59 69^

#### Price discrimination and price-related promotions

Evidence from six recent studies, all from HICs (covering the USA and the UK), highlight how the industry has developed targeted promotional tactics to offer pathways to smokers to reduce their costs.^35 39^ It targets a particular category of consumers and uses price discrimination to lower the price of certain products by using coupons, cartons, gifts, or through differential pricing by geographic location/store type (eg, using retailer rebates). This strategy is beneficial for the industry as it maximises profits by minimising the effect of tax increases on demand and is disproportionately more impactful for the poor or price-sensitive consumers who are more likely to take advantage of price promotions.^72^

In the USA, smokers are able to avoid the full impact of taxes by buying cartons or purchasing from lower tax jurisdictions such as Native American reservations.^32 36^ Tobacco companies have closely monitored ‘border’ or ‘tax-differential’ stores in the country, where they provide extensive promotions of their products to consumers in order to modify shopping patterns and increase sales.^39^ Correspondingly, in the UK between 2002 and 2014, tobacco purchases from supermarkets increased in popularity as the prices were cheaper than those in convenience stores.^45^

#### Price smoothing and shrinkflation

Two studies from the UK identify the use of price smoothing and shrinkflation strategies by the TI. In response to a tax increase every year, the industry was found to initially chose to cut its profits and not (fully) pass on the tax to consumers, thus preventing any quit-inducing large surge in prices but then incrementally increase prices throughout the year in order to slowly pass on the tax increase and thus increase profits.^45^ The extent to which the industry undershifts and the duration over which it increases price was most marked in the lowest price segments and more so in FM than in RYO tobacco.^45^

The TI also masked the increase in prices by keeping pack purchase prices similar to before the tax increase but reduced the size of a standard FM pack (eg, from 20 to 17–19 sticks) and pouch of RYO tobacco (eg, from 12.5 g to 10 g).^42 45^ In the UK, this occurred most markedly in value and subvalue FM categories, where the real pack prices of the cheapest FM and RYO products remained static between 2012 and 2017, despite the rise in price per stick/stick equivalent in all segments.^45^

## Discussion

The primary focus of this study was to assess the extent of literature that exists on TI’s price-based responses to taxation and hence to understand the overall pricing strategies employed worldwide. We can offer no insights into the (relative) success of these countertaxation policies, either in terms of their impact on tax revenue or public health, or if this varies by context, since this was not explored. Overall, the findings strongly suggest that the industry uses a variety of strategies and employs differential tactics for different price tiers of their products, presumably to balance enhancing their revenues/profits with maintaining/boosting the volume of their markets. A large number of studies included in the review were carried out in the USA (11 studies) or the UK (five), and apart from the tactic of differential shifting tax increases, most of the other strategies identified were either more common in (changing product attributes/production processes) or were only found to be present in HICs (price discrimination and promotions, price smoothing, and shrinkflation). This may signify the absence of these strategies in LMICs but may also be a result of their not being considered by the research from these jurisdictions. Hence, the present review highlights the need for more research in this area, most especially in LMICs.^73–75^

Although the predominant pattern in HICs was of overshifting as compared with undershifting in LMICs, not all studies included in the review conformed to this trend; a few demonstrated the presence of both the patterns simultaneously for different cigarette segments,^20 39 47 49 64^ whereas others showed the opposite trend.^52 53^ Therefore, current evidence suggests rate of tax pass-through varies over time and place, as the industry is able to price discriminate by country/income group depending on the tax rate/structure and likely also with the maturity of the market and the extent to which wider tobacco control measures are in place. Another factor that could explain differences is the role of state-owned tobacco monopolies (in markets like China, Vietnam, and Thailand), which face a different set of incentives to privately owned tobacco companies.^76 77^

The TI’s conduct in terms of tobacco tax pass-through can be understood in the context of the global tobacco market, as they balance driving consumption in newer markets with expanding profit margins in mature ones.^55 78^ In HICs, the TI focuses on driving up value rather than volume as a profit maximising model, whereas in many LMICs, the industry pursues the volume expansion model by keeping prices cheap to drive up sales and establish use.^13^ However, in many countries the industry uses a mix of these models, such as in the UK and the USA, where an initial response to tax increases is absorption of taxes on products across all price categories, although premium brands are only undershifted for a short time and are in fact progressively overshifted throughout the rest of the tax-year.^20 45^ Though overshifting is good for reducing tobacco consumption, it represents extra profits for the TI and hence a missed opportunity for governments to have raised taxes even more and therefore collected additional revenues. Certain external factors such as increasing affordability as a result of excise taxes failing to match inflation/growing income levels also play a pivotal role as the industry uses this to its advantage by overshifting taxes as evidenced in a few LMICs, such as Bangladesh and China.^18 20^ This finding is in concordance with another study that showed affordability elasticity as a useful parameter to predict sensitivity of consumers to tobacco tax and price policy changes.^79^ There is also variation with respect to the presence of other tobacco control measures. For example, if legislation is expected to decrease demand, then manufacturers may decide to undershift tax increases at that point in order to reduce the impact of the wider legislature.^1^

A few studies from the review emphasised the popularity of cheaper domestic brands of cigarettes among price sensitive (lower socioeconomic groups and youth) and heavy smokers, particularly in the USA and Mexico.^35 36 56 80^ This finding is in line with other studies on pricing tactics more generally, such as from Canada, where discount and native brands were found to be more popular among youth smokers with relatively less spending money and higher cigarette consumption.^81^ This clear preference for economy brands illustrates why the TI looks to undershift at the lower end of the market.

One interesting point of variation not covered in the highlighted studies is the potential for different companies to respond to taxes in different ways or extents.^82^ We know various companies favour different tax structures (eg, because of their brand portfolios)^83–86^ so these differences might extend to countertaxation strategies too.

The review also illustrates wide disproportions between industry’s claims and actions; the TI aggressively opposes taxation by suggesting it is the cause of the illicit trade (owing to price increases), yet these allegations are inconsistent with their pricing behaviour.^23^ An analysis of the tax and price changes in the UK found that around two-thirds of the increase was due to taxes, while one-third was imposed by the TI.^43^ Furthermore, illicit trade levels are often considerably lower in HICs where prices/taxes on cigarettes are relatively high as opposed to some LMICs, suggesting that price alone does not explain the level of illegal trade. Although illicit is not a focus of our study, the review did not highlight any evidence of increases in taxes influencing the illicit trade, a finding consistent with studies from other LMICs.^87^ This suggests there remains scope for further tax increases in most countries, which should be relatively large and unexpected, as the industry seems better able to adjust its prices to undermine the impact of smaller and/or predictable tax increases.^43 45 78^ Our study also highlighted that in some countries, there may be changes in observed industry pricing strategies over time due to changes in market dynamics. In the case of South Africa, there was a switch from overshifting to undershifting in 2010, which coincided with increased competition from domestic low-cost producers and a rapid increase in the illicit trade.^55 88^ While this might be a unique occurrence, it could also indicate a wider trend and hence needs to be further explored by following industry pricing behaviours over time. Finally, the emergence of heated tobacco products (HTPs) and other nicotine products in recent years have complicated the nature of the tobacco market and may thus have changed industry pricing strategies. Therefore, further research on industry pricing strategies over this full range of products needs to be prioritised.

### Strengths and limitations

We have followed PRISMA reporting standards for systematic reviews. We made extensive attempts to obtain published literature via searches in the five main databases by employing a wide variety of search terms and by including a wide range of study designs in order to avoid overlooking evidence from weaker studies. This review systematically assessed all the available evidence on industry pricing strategies to undermine tobacco excise control interventions/policies, which are relevant to both HICs and LMICs. While the help of a university librarian was sought to pilot and revise the search strategies used herein, there is a possibility that not all relevant studies have been located. There could be other studies that offer some insights on pricing tactics of the industry that have been missed as tax/pricing responses were not their primary focus or they did not report this in their abstracts. Furthermore, we did not search the grey literature and thus may have missed relevant technical reports. However, we believe such missed investigations will be low in number, and hence that we have provided a comprehensive overview of the industry pricing approaches. This is, to our knowledge, the first attempt to provide a broad overview of the global evidence relating to industry efforts to influence excise levels.

The main limitations of this review relate to the quality of included studies as articles were not evaluated for the rigour of their methodology, but rather just for their discussion of industry’s pricing tactics. There is a chance therefore that we may be reporting results obtained from studies of low quality. However, we note that all of the studies were published in peer-reviewed journals, and we found no indications to suggest quality issues during our review process. Nevertheless, methodological differences between the studies might have contributed to the individual findings among the included studies. We are also limited by the evidence available on pricing strategies from LMICs due to the low volume of studies that we found and the relative lack of studies in particular from low-income countries, making it difficult to assess the full response of industry to tax increases in certain jurisdictions or to evaluate the trends across different strategies.

## Conclusion

This systematic review of the global literature on industry’s price-based responses to taxation highlights that the TI use multifaceted strategies to undermine tobacco excise policies all over the world, thereby increasing tobacco consumption and the associated harms. It is not yet clear the extent to which such tactics vary by country or by other local context, as the review also revealed a relative paucity of literature looking at the details of these pricing practices, most especially in LMICs. Further research is therefore needed in this area as these pricing strategies must be routinely monitored and understood so that effective tobacco excise policies can be developed. Given observed industry behaviour, it seems like there is still ample room to increase tobacco taxation in most countries.

**What this paper adds**

**What is already known on this subject**

- Studies covering tobacco industry price-based responses to tobacco excise tax policies exist, but there has been no attempt to collate or collectively assess this information, including how it varies by country/context.

**What this paper adds**

- This paper systematically reviews current evidence of tobacco industry pricing strategies to undermine tobacco tax policies.
- The review determines that tobacco industry tends to employ a variety of pricing tactics to mitigate the positive tobacco control effects of tax increases and that the six most commonly identified strategies in the existing literature both from high-income and low-income and middle income countries are: differential shifting of tax increases; shrinkflation; price smoothing; changing product attributes and production processes; introducing new brands/segments/products as pathways of downtrade for consumers; and price discrimination and price-related promotions.
- The review highlights the paucity of research on tobacco industry pricing efforts to undermine taxation especially from low-income and middle-income countries.
